# Investigating Distinct Skin Microbial Communities and Skin Metabolome Profiles in Atopic Dermatitis

**DOI:** 10.3390/ijms25105211

**Published:** 2024-05-10

**Authors:** Suyeon Kim, Minah Cho, Eun Sung Jung, Inseon Sim, Yu Ri Woo

**Affiliations:** 1 Department of Dermatology, Incheon St. Mary’s Hospital, College of Medicine, The Catholic University of Korea, Seoul 06591, Republic of Korea; 2 HEM Pharma Inc., Suwon 16229, Gyeonggi, Republic of Korea; esjung@hempharma.bio (E.S.J.);

**Keywords:** atopic dermatitis, atopic eczema, skin, microbiota, metabolite

## Abstract

Atopic dermatitis (AD) is a chronic inflammatory skin disorder influenced by genetic predisposition, environmental factors, immune dysregulation, and skin barrier dysfunction. The skin microbiome and metabolome play crucial roles in modulating the skin’s immune environment and integrity. However, their specific contributions to AD remain unclear. We aimed to investigate the distinct skin microbial communities and skin metabolic compounds in AD patients compared to healthy controls (HCs). Seven patients with AD patients and seven HCs were enrolled, from whom skin samples were obtained for examination. The study involved 16S rRNA metagenomic sequencing and bioinformatics analysis as well as the use of gas chromatography time-of-flight mass spectrometry (GC-TOF-MS) to detect metabolites associated with AD in the skin. We observed significant differences in microbial diversity between lesional and non-lesional skin of AD patients and HCs. *Staphylococcus* overgrowth was prominent in AD lesions, while *Cutibacterium* levels were decreased. Metabolomic analysis revealed elevated levels of several metabolites, including hypoxanthine and glycerol-3-phosphate in AD lesions, indicating perturbations in purine metabolism and energy production pathways. Moreover, we found a positive correlation between hypoxanthine and glycerol-3-phosphate and clinical severity of AD and *Staphylococcus* overgrowth. These findings suggest potential biomarkers for monitoring AD severity. Further research is needed to elucidate the causal relationships between microbial dysbiosis, metabolic alterations, and AD progression, paving the way for targeted therapeutic interventions.

## 1. Introduction

Atopic dermatitis (AD) is a chronic, relapsing inflammatory skin disorder characterized by intense pruritus, xerosis, and recurrent eczematous skin lesions. It is a multifactorial disease influenced by genetic predisposition, environmental factors, immune dysregulation, and skin barrier dysfunction. Among the many factors contributing to AD, the skin microbiome and metabolome play pivotal roles in modulating the skin’s immune environment and integrity.

The skin microbiome refers to the diverse community of microorganisms, including bacteria, fungi, and viruses, residing on the skin surface [1]. These microbial communities are crucial for maintaining skin health, protecting against pathogens, and modulating immune responses. In AD, dysbiosis of the skin microbiome, particularly the overgrowth of *Staphylococcus aureus* (*S. aureus*), has been closely associated with disease severity [2,3]. 

The skin metabolome encompasses the complete set of small-molecule chemicals found within the skin, reflecting the metabolic processes that occur in response to both internal and external stimuli. Metabolites, which are small molecules generated through cellular activities, serve as indicators of metabolic functions and conditions related to diseases.

Recent insights underscore the intricate interplay between the skin microbiome, neuroendocrine signaling, and immune responses in the context of AD. Studies have elucidated communication pathways between the epidermal microbiome and the epidermal neuroendocrine network, shedding light on their roles in modulating skin physiology and immune functions [4,5]. Despite advancements, gaps persist in the understanding of how the skin microbiome and metabolome interact in AD.

Understanding how the metabolome affects the microbiome and vice versa is essential for a deeper understanding of AD. Some previous studies have investigated how the skin’s microbiota and metabolites interact in AD [6,7,8,9]. Skin microbes could alter the skin’s metabolomic profile by producing enzymes that metabolize host lipids, proteins, antimicrobial peptides, and macromolecules, thereby impacting skin barrier integrity and immune responses [10]. Conversely, Emmert et al. [9] reported an increase in free fatty acids (FFA) in AD skin, and this was largely associated with *Staphylococcus*. There is a noted inverse relationship between the levels of saturated, shorter-chain FFAs and the presence of *Staphylococcus* [9], as shorter fatty acids can penetrate and acidify the skin, inhibiting *S. aureus* growth, which prefers less-acidic conditions [10]. Yet, there is limited knowledge regarding the specific changes in metabolites beyond skin lipids and skin metabolites or how these metabolites relate to the skin microbiome.

This study aims to investigate the distinct microbial communities and metabolic compounds in patients with AD. By comparing the skin microbial and skin metabolic profiles of lesional skin and non-lesional skin of AD and to those of healthy controls (HCs), we intended to seek to unravel the specific contributions of these factors to the pathophysiology of AD. Moreover, this study intends to establish connections between the composition of the skin microbiota, skin metabolic profiles, and AD’s clinical characteristics to pinpoint potential biomarkers for AD. Understanding these relationships is crucial for developing targeted therapeutic strategies and improving the management of AD. Through this study, we aspire to contribute to the broader understanding of AD’s underlying mechanisms and pave the way for novel interventions that address the microbial and metabolic dimensions of this complex skin disorder.

## 2. Results

### 2.1. Demographic Characteristics

The participants’ clinical characteristics are delineated in Supplementary Table S1. This study included a total of seven individuals with AD and seven HCs. Comparative analysis yielded no substantial disparities in age, sex, or body mass index (BMI) between the AD cohort and the HC group. Notably, the AD cohort manifested significantly elevated Eczema Area and Severity Index (EASI) scores, serum eosinophil percentages, total immunoglobulin E (IgE) levels compared to the HCs. While the pH of lesional epidermis in the AD patients was found to be increased relative to their non-lesional counterparts and the skin of the HCs, this variation did not achieve statistical significance. Furthermore, there was a significant difference in transepidermal water loss (TEWL) and the erythema index when comparing the lesional and non-lesional skin of AD and the skin of the HCs.

The dataset encompassed 21 specimens analyzed through bacterial 16S rDNA V3-V4 sequencing. The resulting high-fidelity reads totaled an average count of 183,122, as outlined in Supplementary Table S2. Sequences were clustered at a 99% similarity threshold, culminating in the identification of a comprehensive array of amplicon sequence variants (ASVs) that represent the bacterial microbiota.

### 2.2. Diversity Analysis

**Figure 1:**
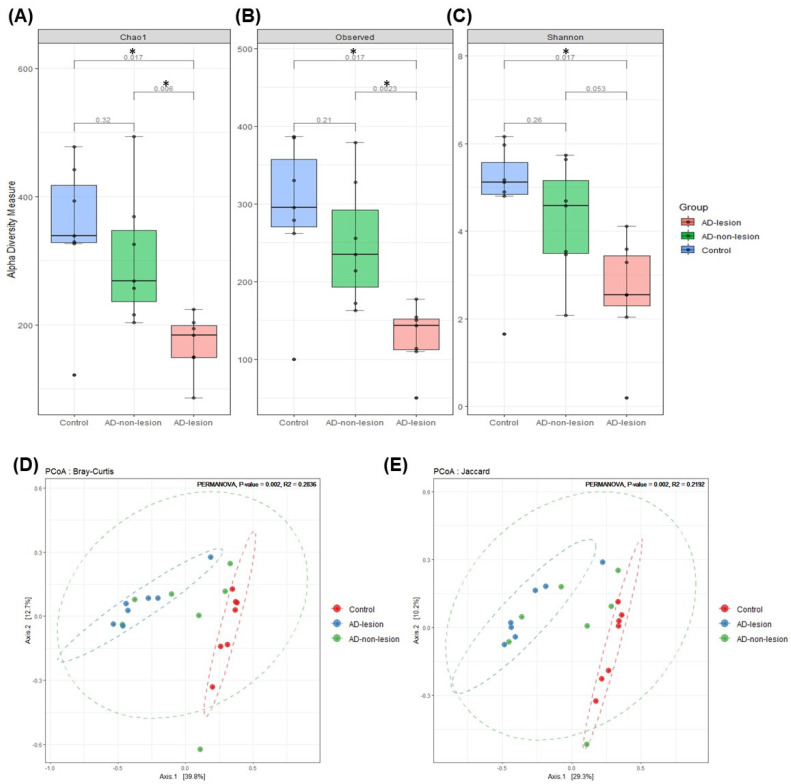
Bacterial diversity in the patients with atopic dermatitis and healthy controls. (**A**–**C**) Alpha diversity of bacterial taxa, analyzed using the Chao1, Observed, and Shannon indices, respectively. (**D**,**E**) Two-dimensional principal coordinate analysis (PCoA) plot depicting bacterial taxa diversity, calculated using the Bray–Curtis and Jaccard indices. The asterisk (*) represents the significant level: * *p*< 0.05.

To assess the α diversity of each sample, we utilized the Chao1, Observed, and Shannon indices. The Chao1 index, which indicates the richness of the bacterial population, and the Observed index, reflecting the number of species distributed per sample, exhibited significantly lower values in the lesional skin of the AD group compared to the non-lesional AD group and the HCs (Figure 1A,B). The Shannon index, which represents both the evenness and abundance of the bacterial population, showed significantly lower values in the lesional skin of AD compared to the HCs (Figure 1C). However, the difference between the lesional skin of the AD group and the non-lesional skin of the AD group was not statistically significant (Figure 1C).

We observed specific clustering within the microbial community between the groups using the Bray–Curtis and Jaccard indices (Figure 1D). PERMANOVA analysis revealed statistically significant variations in beta diversity when comparing among the lesional skin of the AD group, the non-lesional skin of the AD group, and the HCs (Bray–Curtis: *p* = 0.002, R2 = 0.283; Jaccard: *p* = 0.002, R2 = 0.219).

### 2.3. Taxonomic Composition of Bacterial Microbiota on the AD Skin

**Figure 2:**
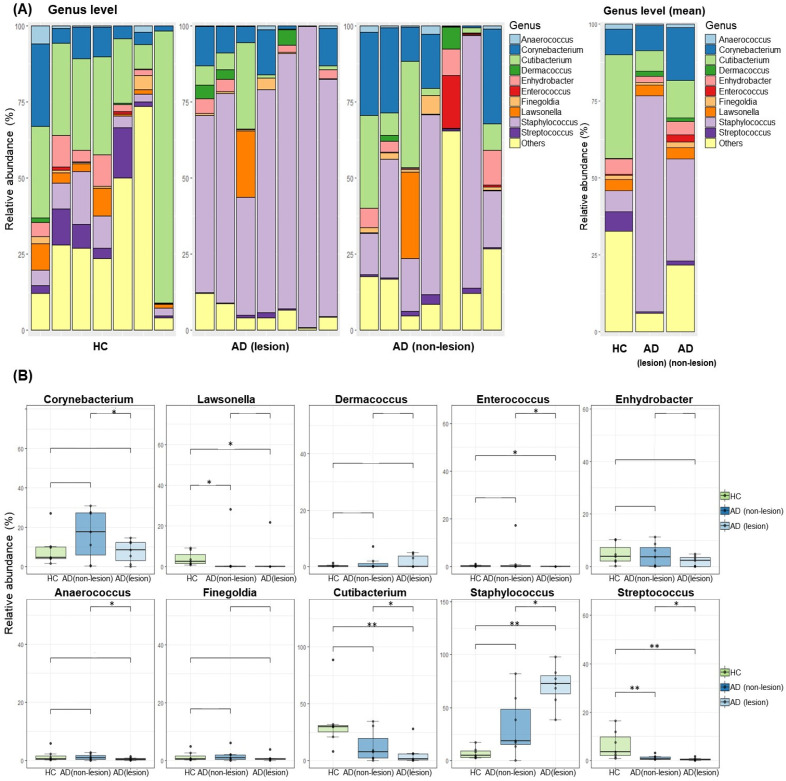
Skin microbiota composition in the lesional and non-lesional skin of patients with atopic dermatitis (AD) and healthy controls (HCs). (**A**) Relative abundance of the major bacterial genera in the lesional and non-lesional skin of AD and in HCs. (**B**) A plot depicting bacteria at the genus level with significant differences in relative abundance between the AD and HC groups. The asterisks (*,**) represent the significant levels: *, *p*< 0.05; **, *p* < 0.01.

A comprehensive analysis of the bacterial microbiota on the skin revealed the presence of 27 bacterial phyla, with Firmicutes (50.04%), Actinobacteriota (38.16%), Proteobacteria (8.60%), and Bacteroidota (2.12%) being the most predominant. In our study, we identified 600 bacterial genera on the skin. The ten most abundant bacterial genera in all the skin specimens were *Staphylococcus*, *Cutibacterium*, *Corynebacterium*, *Enhydrobacter*, *Lawsonella*, *Streptococcus*, *Finegoldia*, *Dermacoccus*, *Anaerococcus*, and *Enterococcus* (Figure 2A). The ten most abundant bacterial genera in the AD patients’ skin were *Staphylococcus*, *Corynebacterium*, *Cutibacterium*, *Lawsonella*, *Enhydrobacter*, *Dermacoccus*, *Finegoldia*, *Enterococcus*, *Streoptococcus*, and *Bacteroides.* Notably, the lesional skin of AD showed an increased relative abundance of *Staphylococcus* (*p* = 0.002) than the HCs. In addition, the lesional skin of AD showed an increased relative abundance of *Staphylococcus* than the non-lesional skin of AD (*p* = 0.015). The lesional skin of AD showed a decreased abundance of *Cutibacterium* (*p* = 0.007), *Streptococcus* (*p* = 0.012), *Enterococcus* (*p* = 0.039), and *Lawsonella* (*p* = 0.039) than the HCs (Figure 2B). In addition, a statistically significant decrease in the mean relative abundance of *Cutibacterium* (*p* = 0.015), *Streptococcus* (*p* = 0.015), *Enterococcus* (*p* = 0.015), *Corynebacterium* (*p* = 0.046), and *Anaerococcus* (*p* = 0.046) was observed in the lesional skin of AD when compared to the non-lesional skin of AD (Figure 2B).

### 2.4. Differential Skin Metabolic Profiles in Patients with AD Compared to HCs

**Figure 3:**
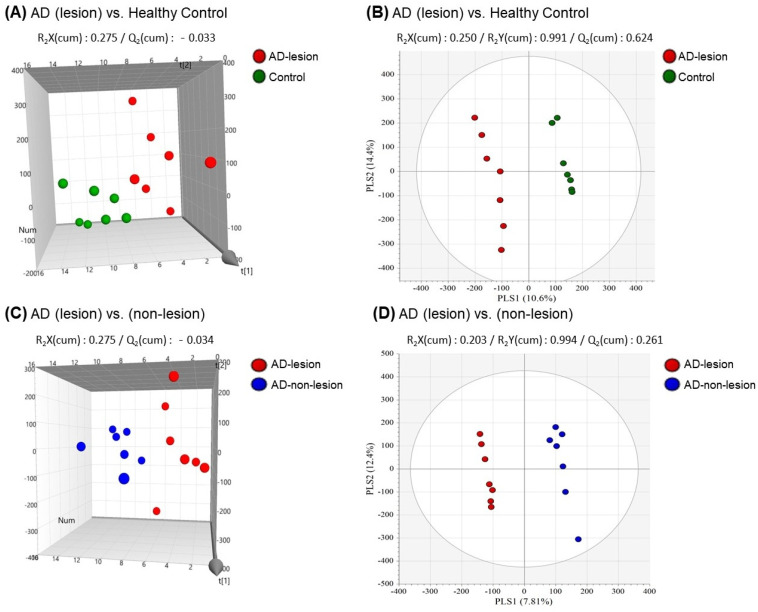
Differentiation between patients with atopic dermatitis (AD) and healthy controls (HCs) using metabolic profiles. (**A**) Scatterplot of 3D principal component analysis (PCA) model between lesional skin of AD and HCs. (**B**) Scatterplot of partial least squares discriminant analysis (PLS-DA) between lesional skin of AD and HCs. (**C**) Scatterplot of 3D PCA model between lesional skin of AD and non-lesional skin of AD. (**D**) Scatterplot of PLS-DA between lesional skin of AD and non-lesional skin of AD.

To distinguish the metabolic profiles between the lesional and non-lesional skin of AD and the HCs, we used principal component analysis (PCA) and partial least squares discriminant analysis (PLS-DA). The PCA plot showed the separation between AD (lesional skin) and HCs (R2X = 0.275, Q2 = −0.033, Figure 3A). Additionally, the PLS-DA score plot confirmed a distinct separation among the lesional skin of AD and HCs (R2X = 0.250, R2Y = 0.991, Q2 = 0.624, Figure 3B). When comparing the lesional skin of AD to the non-lesional skin of AD, the PCA results demonstrated a clear distinction between the AD lesional skin and the AD non-lesional skin, with the PCA plot indicating a separation (R2X = 0.275, Q2 = −0.034, Figure 3C). Further analysis using the PLS-DA score plot also highlighted a clear differentiation between the metabolic profiles of the lesional and non-lesional skin of AD, showcasing distinct clusters for each group (R2X = 0.203, R2Y = 0.994, Q2 = 0.261, Figure 3D).

**Figure 4:**
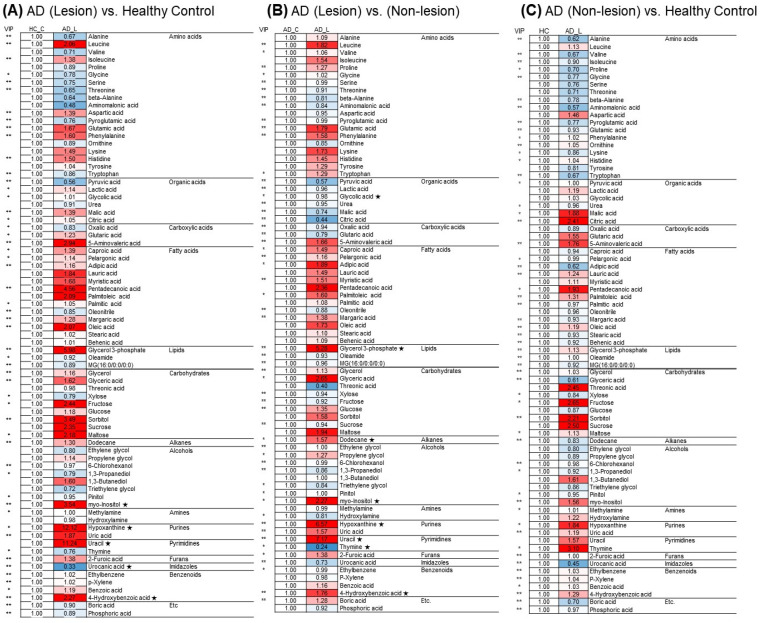
(**A**) A heatmap illustrating the differences in skin metabolite levels between the lesional skin of atopic dermatitis (AD) and healthy controls (HCs). (**B**) A heatmap revealing the variations in metabolite levels between lesional and non-lesional skin of AD patients. (**C**) A heatmap revealing the variations in metabolite levels between non-lesional skin of AD patients and HCs. ‘*’ indicates variable importance in projection (VIP) values exceeding 0.7, ‘**’ indicates VIP values exceeding 1.0, and ‘★’ indicates Kruskal–Wallis’s rank-sum tests with false-discovery-rate-corrected *p*-values less than 0.05.

We screened for differential metabolites using the first principal component of variable importance in the projection (VIP). Metabolites with VIP values exceeding 0.7 and Kruskal–Wallis’s rank-sum tests with false discovery rate (FDR)-corrected *p*-values less than 0.05 were identified. A comparison of metabolite levels between lesional AD patients and HCs revealed increased levels of four metabolites, including hypoxanthine, myo-inositol, uracil, and 4-hydroxybenzoic acid, along with a decreased level of one metabolite, namely urocanic acid (Figure 4A).

A pairwise comparison of metabolite levels between lesional and non-lesional skin utilized the same VIP criteria and a *p*-value of less than 0.05 in the Mann–Whitney U test. In the lesional AD skin, compared to non-lesional AD skin, six metabolites were found to be increased (uracil, hypoxanthine, glycerol-3-phosphate, myo-inositol, 4-hydroxybenzoic acid, and dodecane), and two metabolites were found to be decreased (thymine and glycolic acid), as shown in Figure 4B.

### 2.5. Associations between Skin Metabolites with Skin Microbiota and Clinical Factors in AD Patients

**Figure 5:**
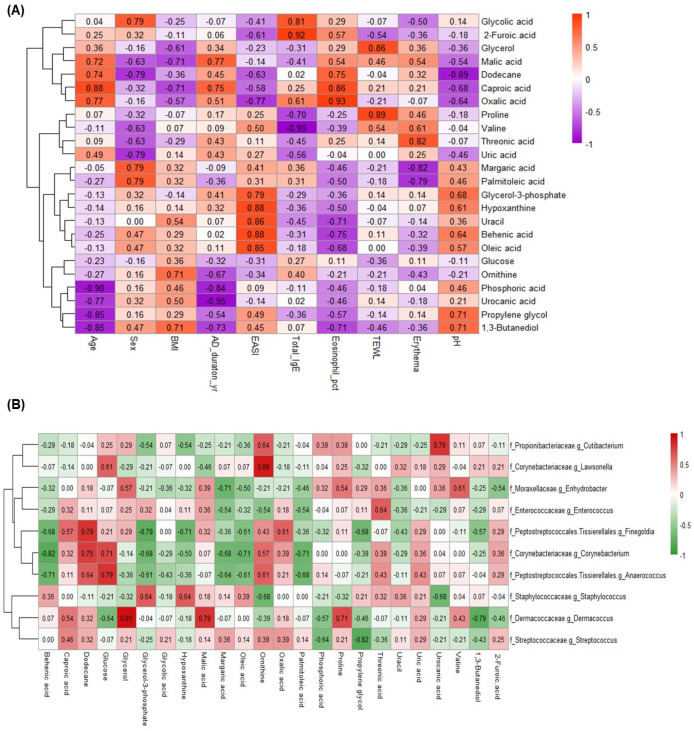
Associations between skin metabolites, clinical parameters, and microbial diversity in AD skin lesions. (**A**) A heatmap generated from Spearman’s correlation analysis illustrating the relationships between skin metabolites and various clinical parameters in lesional skin of AD. (**B**) A heatmap from Spearman’s correlation analysis depicting the correlations between skin metabolites and the composition of skin microbiota in lesional skin of AD.

To investigate the relationships among skin metabolites, the biophysical characteristics of skin, and the severity of AD, we performed correlation analyses. We then visualized these relationships in a heatmap, focusing on Spearman’s correlation coefficients greater than 0.6 or less than −0.6, with significance defined by *p*-values below 0.05 (Figure 5A). The EASI score showed significant positive correlations with several metabolites, including hypoxanthine, behenic acid, uracil, oleic acid, and glycerol-3-phosphate, but negative correlations with 2-furoic acid, dodecane, and oxalic acid (all *p* < 0.05). Similarly, the skin pH in the lesional skin correlated positively with metabolites such as glycerol-3-phosphate, and hypoxanthine and negatively with dodecane, caproic acid, and oxalic acid.

### 2.6. Associations between Skin Metabolites and Skin Microbiota

As shown in Figure 5B, our analysis uncovered significant relationships between certain bacterial groups and metabolites. In particular, *Staphylococcus* showed a positive correlation with glycerol-3-phosphate and hypoxanthine and a negative correlation with ornithine and urocanic acid. In addition, glycerol-3-phosphate showed a negative correlation with *Finegoldia, Corynebacterium*, and *Anaerococcus*.

*Cutibacterium* exhibited notable positive correlations with ornithine and urocanic acid. *Lawsonella* exhibited significant positive correlations with glucose and ornithine. Several other prominent bacterial genera, including *Corynebacterium*, *Enhydrobacter*, *Streptococcus*, *Finegoldia*, *Dermacoccus*, *Anaerococcus*, and *Enterococcus*, were found to have significant correlations with a variety of metabolites.

## 3. Discussion

We analyzed the bacterial microbiota in skin lesions from individuals with AD and compared them to both non-lesional skin from the same AD patients and the skin samples from HCs. Distinct variations in the bacterial populations in the skin were identified across these groups. Additionally, we explored the unique metabolomic profiles of the skin of AD patients, uncovering significant differences in metabolite expression between the lesional and non-lesional skin of AD as well as compared to that of the HCs.

This study observed a significant reduction in the diversity indices in lesional skin of AD patients compared to the non-lesional skin of AD and HCs, suggesting a less diverse bacterial community in AD, which is consistent with previous findings [11,12]. The significant reduction in microbial diversity, particularly in the lesional skin of the AD patients, underscores the critical role of the skin microbiome in maintaining cutaneous health and its potential disruption in disease states. Studies have shown that a diverse microbial community contributes to skin barrier integrity, immune regulation, and protection against pathogenic colonization.

We found an increased relative abundance of *Staphylococcus* in the lesional skin of AD compared to both the non-lesional skin of AD and HCs. Moreover, there was a decreased relative abundance of *Cutibacterium* and *Streptococcus* in the lesional skin of AD when compared to both the non-lesional skin of AD patients and HCs, respectively. The dominance of *Staphylococcus*, especially *S. aureus*, in AD lesions has been well documented and is known to exacerbate the inflammatory response, leading to a worsening of AD symptoms [11]. AD patients colonized by *S. aureus* exhibit a higher degree of Th2 immune response polarization, increased sensitivity to allergens, and more extensive tissue damage in comparison to individuals who are not colonized [13]. The decrease in beneficial microbes, such as *Cutibacterium*, which are involved in the production of short-chain fatty acids [14,15], further exacerbates skin barrier dysfunction. In addition, we found that the skin of AD patients is already compromised, regardless of the presence of AD lesions. This observation is supported by Figure 2A, which shows that the non-lesional skin microbiota of AD patients does not resemble that of HCs. There is already a detectable difference in the skin microbiota with patients with AD compared to HCs.

The metabolic profiling presented in this study, showing distinct differences between the lesional and non-lesional skin of AD patients and HCs, highlights the profound impact of AD on skin metabolism. Metabolomics provides a detailed method for examining metabolites present in a wide range of biological materials, such as cells, tissues, and fluids, in order to identify how alterations in these metabolites relate to health or disease conditions [16]. The analysis often involves biofluids like serum, plasma, urine, saliva, tears, cerebrospinal fluid, bile, pancreatic juice, intestinal fluid, and breast milk, in addition to cellular and tissue samples [16]. Skin samples, in particular, offer crucial insights into the metabolomic profile associated with skin diseases, extending beyond the conventional analysis of blood and urine. Different biological specimens can reveal unique information. For instance, analyzing the skin surface can significantly improve our understanding of metabolites linked to skin conditions. A range of skin sampling methods, from invasive procedures like biopsies to non-invasive approaches such as skin swabbing, tape stripping, hydrogel micropatches, wet prep, and suction blisters have been established [17]. A study has demonstrated that tape stripping excels in gathering a wider range of metabolites when compared to the swabbing and wet prep methods [17]. By conducting non-invasive tape stripping, we performed the skin metabolomic analysis in the patients with AD.

In our study, we observed significant metabolic changes in the lipid and purine pathways in AD. Specifically, hypoxanthine levels were higher in AD lesions compared to both non-lesioned AD skin and HCs, suggesting an upregulation of purine metabolism possibly linked to increased DNA repair and replication in response to skin damage. Hypoxanthine, a fundamental component of nucleic acids, plays a crucial role in energy transactions and cellular signaling [18]. Through the purine salvage pathway, hypoxanthine is recycled into nucleotides essential for DNA repair and other cellular functions [18]. The elevated hypoxanthine in AD could be explained by the fast turnover and damage in AD skin necessitating increased purine metabolism for recycling cell components. In addition, the chronic skin inflammation characteristic of AD may prompt immune cells to release substances that break down nucleotides to purines such as hypoxanthine. Similarly, a recent study also observed elevated serum levels of hypoxanthine in asthma patients [19]. This increase in hypoxanthine among asthma patients might be associated with inhibited xanthine oxidase activity, or it could result from an accumulation of hypoxanthine in the purine metabolic pathway linked to asthma [19].

In addition, this study also found that there was an increased level of glycerol-3-phosphate in the lesional skin of AD compared to that of the non-lesional skin of AD. Glycerol-3-phosphate is a crucial element in energy production processes such as glycolysis and the formation of glycerolipids [20]. It is involved in regulating metabolic pathways, especially the cycle of glycerolipids and free fatty acids [21,22]. It also forms the core structure for triglyceride synthesis by combining with three fatty acyl-CoAs [21,22]. Excessive glycerol-3-phosphate may cause metabolic stress, increase reactive oxygen species, and damage essential biomolecules [21,22]. As glycerol-3-phosphate is crucial for energy production and lipid synthesis, it is speculated that this could exacerbate AD by leading to cellular damage, skin barrier dysfunction, and persistent inflammation in AD.

In addition, we found an imbalance in nitrogen (N) metabolism in the AD patients. The nitrogen (N) imbalance is suggested by the insufficient turnover of amino acids and elevated levels of purines and thymine-related metabolites. These metabolites are typically associated with DNA repair and synthesis, yet their altered levels point to a disruption in nitrogen metabolism. Intriguingly, this imbalance in nitrogen metabolism cannot be corrected by the already compromised skin microbiome of AD patients. The nitrogen imbalance was indicated by an inadequate turnover of amino acids and heightened levels of purines and thymine-related metabolites in our study. These metabolites are typically involved in DNA repair and synthesis, further implying a disrupted nitrogen metabolism.

We observed a positive correlation between hypoxanthine and glycerol-3-phosphate levels with AD’s clinical severity. Additionally, a negative correlation was observed between mean relative abundance of *Staphylococcus* and the levels of hypoxanthine and glycerol-3-phosphate in the lesional skin of AD. This suggests a potential link between the clinical severity of AD and the levels of certain metabolites, indicating that the microbial and metabolic profiles could be integral to understanding and treating AD more effectively. The integration of the microbiome, metabolome, and clinical data provides a deeper insight into AD’s pathophysiology, laying the groundwork for personalized treatments.

Acknowledging the limitations of this study, such as its small sample size and single-center cohort design, underscores the need for large-scale validation studies to ensure reliability and applicability globally. Moreover, while promising, the translation of insights from microbiome and metabolome analyses into clinical practice faces challenges. The variability in microbial and metabolic profiles among individuals, influenced by genetics, environment, and lifestyle, complicates the identification of universal biomarkers or therapeutic targets. Additionally, the dynamic nature of the microbiome and metabolome necessitates longitudinal studies to capture fluctuations and their implications for disease management. Future research should aim to elucidate causal relationships through longitudinal and interventional studies that target the impact of the microbiome and metabolome on disease outcomes.

In conclusion, our study highlights the significant role of the skin microbiome and skin metabolome in AD. We found distinct variations in bacterial populations and metabolic profiles between the lesional and non-lesional skin of AD patients and HCs, indicating microbial dysbiosis and metabolic alterations associated with AD. We observed significant differences in microbial diversity between the lesional and non-lesional skin of AD patients and HCs, with a notable overgrowth of *Staphylococcus* in AD lesions, while the abundance of *Cutibacterium* was decreased. These variations indicate microbial dysbiosis and suggest a specific microbial signature associated with the disease state. Additionally, our metabolomic analysis revealed elevated levels of hypoxanthine and glycerol-3-phosphate in AD lesions, suggesting perturbations in purine metabolism and energy production pathways. These findings suggest potential biomarkers for monitoring AD severity and underscore the complex interplay between microbial and metabolic factors in the disease’s pathogenesis. Further research is needed to elucidate causal relationships between microbial dysbiosis, metabolic alterations, and AD progression, leading the way for targeted therapeutic interventions.

## 4. Materials and Methods

### 4.1. Recruitment of Study Participants

This study involved adult patients with AD, as confirmed by board-certified dermatologists, and compared them to HCs without AD. Recruitment took place at Incheon St. Mary’s Hospital, Republic of Korea, from September 2022 to March 2023. Participants under 18, those who had taken antibiotics or antifungal treatments in the past month, and those with a history of skin cancer were excluded. Written informed consent was obtained from all participants prior to their involvement in the study. The research investigation adhered to the ethical principles outlined in the Declaration of Helsinki and received approval from the ethics committee of Incheon St. Mary’s Hospital (OC22TISI0088).

### 4.2. Skin Sample Collection

The collection of skin samples was conducted from lesional and non-lesional normal-appearing antecubital skin located 5–10 cm apart in patients with AD and antecubital skin lesions from HCs. Skin swabbing was performed for microbiota sampling from the skin (a single sampling covering a 4 cm^2^ area) using sterile cotton swabs (Transportsystem^TM^ 108C, Copan Diagnostics Inc., Murrieta, CA, USA). Tape strip sampling was also conducted to sample the metabolites from the skin using D-Squame^®^ standard sampling discs (Cuderm Corporation, Dallas, TX, USA). The swabs and tape strips were kept at a temperature of −80 °C until further analysis.

The biophysical parameters of the affected skin were evaluated by measuring the quantity of TEWL, skin sebum, pH levels, and the erythema index. A tewameter TM HEX, sebumeter SM825, pH meter PH900, and a mexameter MX16 (manufactured by Courage + Khazaka Electronic GmbH, Cologne, Germany) were used for these assessments. Each parameter was measured three times per probe, and the average value was calculated for analysis. The severity of AD was evaluated by a board-certified dermatologist using the EASI scores [23].

### 4.3. DNA Extraction and Next Generation Sequencing (NGS) Analysis

Bacterial DNA extraction from the skin swab samples was performed using a Mag-Bind^®^ Universal Pathogen Kit. This involved suspending the samples in SLX-Mlus Buffer and bead beating, followed by purification as per the manufacturer’s protocols (Omega Bio-tek, Norcross, GA, USA).

The V3-V4 regions of the 16S rRNA gene were amplified for bacterial identification using specific primers, as described in the manufacturer’s guidelines. This included incorporating multiplexing indices and Illumina sequencing adapters (Illumina Inc., San Diego, CA, USA). Post amplification, the samples were barcoded, and library sizes were verified using LabChip (PerkinElmer, Waltham, MA, USA) and a QubitTM 1X dsDNA BR Assay Kit (Thermo Fisher Scientific, Waltham, MA, USA). Sequencing was conducted on an Illumina Miseq platform, ensuring anonymity of the original sequencing reads.

Subsequent data processing included quality filtering, demultiplexing, and denoising using QIIME2 2021.2 and DADA2 [24], with taxonomic classification being conducted via Silva138 [25]. The analyses were conducted using the phyloseq package in the R software (version 4.3.0).

The alpha diversity indices such as Shannon, Observed, and Chao1 measured the average species diversity within the samples. Beta diversity was assessed using the Bray–Curtis and Jaccard indices, with PCoA and PERMANOVA (999 permutations) used for analyzing group variations. Statistical comparisons among groups employed the non-parametric Kruskal–Wallis and Wilcoxon rank tests, with *p*-values adjusted for multiple testing via the Benjamini–Hochberg method to maintain an FDR of 0.05.

### 4.4. Sample Extraction for Metabolomic Analysis and GC-TOF Analysis

For metabolite extraction from AD patients, three tape strips per sample were cut, mixed with 1 mL of methanol, vortexed, and sonicated. The extracted samples were then mixed with an internal standard (2-chloro-phenylalanine) and dried using a centrifugal vacuum concentrator (HyperVAC, Daejeon, Republic of Korea). The dried samples underwent derivatization with methoxyamine hydrochloride and N-methyl-N-trimethylsilyl-trifluoroacetamide (MSTFA) followed by filtering for analysis.

GC-TOF-MS analysis was conducted on an Agilent 7890 system (Agilent Technologies, Palo Alto, CA, USA) with a Pegasus BT-TOF-MS. The process involved using an Rtx-5MS column, helium carrier gas, and specific temperature settings. Electro-ionization and full scanning were performed for data collection. The obtained data were pre-processed using the Chroma TOF software (LECO corporation, version 5.51.50.0) and aligned using the Metalign software (version 041012). Multivariate statistical analysis, including PCA, PLS-DA, and OPLS-DA, was performed using the SIMCA P+ software (version 16.0, Umetrics, Umea, Sweden) to analyze the metabolites.

## Data Availability

Data is contained within the article.

## Supplementary Materials

The following supporting information can be downloaded at: https://www.mdpi.com/article/10.3390/ijms25105211/s1.
