# Supplementary material for: Investigating Distinct Skin Microbial Communities and Skin Metabolome Profiles in Atopic Dermatitis
Source: Int J Mol Sci. 2024 May 10;25(10):5211. doi: 10.3390/ijms25105211 (PMC11121500; doi:10.3390/ijms25105211)
Supplement: Supplementary file 1 [file ijms-25-05211-s001.zip › ijms-2950061-supplementary.pdf]

Table S1. Demographic and clinical characteristics.

	AD (n = 7)		Healthy control (n = 7)	P-value
Age	28.29 ± 7.41		29.57 ± 2.82	0.68
Sex				1.00
Male	5 (71.42)		5 (71.42)	
Female	2 (28.58)		2 (28.58)	
BMI (kg/m^2)	26.06 ± 2.05		22.95 ± 1.17	0.21
EASI	24.31 ± 3.91		0	<0.001
Eosinophil (%)	6.74 ± 1.48		1.02 ± 0.15	0.008
Total IgE	1246.57 ± 350.29		33.14 ± 6.47	0.013
	Lesional skin	Non-lesional skin		
TEWL	32.14 ± 2.88	19.31 ± 3.62	12.22 ± 2.54	<0.001
Erythema index (AU)	396.66 ± 54.57	229.33 ± 44.09	183.95 ± 28.92	0.007
pH	5.72 ± 0.26	5.45 ± 1.99	5.32 ± 0.10	0.359

Data are expressed as the mean ± standard deviation or number (percentage). Abbreviations: AD, atopic dermatitis; AU, arbitrary unit; EASI, eczema area severity index; HC, healthy control; IGA, investigators global assessment; NRS, numerical rating scale.

Table S2. Summary of total read counts for sequence reads.

Group/Sample No.	Total read count
AD-L-2-S001	179,624
AD-L-7-S001	147,778
AD-L-6-S001	214,274
AD-L-9-S001	174,120
AD-L-10-S001	160,274
AD-L-11-S001	179,486
AD-L-14-S001	195,670
AD-C-2-S001	206,386
AD-C-6-S001	174,288
AD-C-7-S001	157,710
AD-C-9-S001	192,112
AD-C-10-S001	205,328
AD-C-11-S001	163,698
AD-C-14-S001	171,130
HC-C-1-S001	181,880
HC-C-4-S001	164,308
HC-C-5-S001	151,156
HC-C-7-S001	160,524
HC-C-8-S001	203,744
HC-C-9-S001	185,980
HC-C-10-S001	276,084
